# Association between Ultra-Processed Food Consumption and Diabetes in Chinese Adults—Results from the China Health and Nutrition Survey

**DOI:** 10.3390/nu14204241

**Published:** 2022-10-12

**Authors:** Ming Li, Zumin Shi

**Affiliations:** 1 Centre for Population Health Research, Division of Health Sciences, University of South Australia, Adelaide, SA 5000, Australia; 2 Human Nutrition Department, College of Health Sciences, QU Health, Qatar University, Doha 2713, Qatar

**Keywords:** ultra-processed food, long-term consumption, diabetes, China, adults

## Abstract

Aims: We aimed to assess the association between ultra-processed food (UPF) consumption with diabetes in Chinese adults. Methods: This study included 12,849 eligible adults aged 20 years and over attending at least two surveys in the China Nutrition and Health Survey during 1997–2011. Food intake at each survey was assessed by a 3-day 24-h dietary recall method. UPF was defined based on the NOVA classification. Diabetes was obtained from questionnaires and/or ascertained by fasting blood tests. The association of diabetes with UPF was examined using mix effect logistic regression adjusting for potential confounding factors. Results: The mean age of the participants was 43.3 (SD 14.8) years. The age and gender adjusted mean UPF intake increased four times and the prevalence of diabetes increased eight times in 1997–2011. Compared with non-consumers, the odds ratios (95% CI) of diabetes for those with mean UPF consumption of 1–19 g/day, 20–49 g/day, and ≥50 g/day were 1.21 (0.98, 1.48), 1.49 (1.19, 1.86), and 1.40 (1.08, 1.80), respectively (p trend < 0.001) after adjusted for the measured covariates including lifestyle factors (smoking, alcohol drinking, and physical activity), BMI and hypertension. Conclusions: both UPF consumption and prevalence of diabetes increased among adults in China during 1997–2011. Higher UPF consumption was positively associated with diabetes.

## 1. Introduction

Diabetes is a global health issue contributing to many severe complications and posing huge economic burden [1]. It affected 10.5% in 20–79 years old of the general population worldwide and China has the most people with diabetes with estimates of over 140 million in 2021 with projections of 174.4 million in 2045 [2]. In addition to the known risk factors including overweight/obesity, sedentary lifestyle, family history, hypertension, and elevated levels of triglycerides, diet attributed to 34.9% of disability-adjusted life years of diabetes [3], such that processed meat, refined grains, and fried products were positively associated with diabetes [4].

The classifications based solely on nutrient composition failed to explain the entire influence of food consumption on diabetes [5]. NOVA classifies foods and drinks into four groups based on food processing and brings a perspective insight into the diabetes epidemic [6]. Ultra-processed food (UPF) is the 4th Group in NOVA including products of entirely industrial formulations or made from substances extracted from foods, with minimal whole foods [6]. UPF is commonly high in energy density, sugars, salt, trans fats as well as additives, but low in protein, micronutrients, and fibers. UPF takes up more than half of total daily energy intake in high-income countries and its consumption is increasing rapidly in middle-income countries [7]. 

Accumulating evidence have indicated an adverse impact of high UPF intake on metabolic health, including cardiovascular diseases and mortality [8,9]. The evidence from the animal experiment indicates that UPF is a significant risk factor hyperinsulinemia and glucose intolerance [10], and certain types of UPF (e.g., soda and processed meats) were correlated with diabetes [11,12]. A recent meta-analysis of five observational studies from France, Netherland, Spain, UK, and Canada indicated each 10% increase UPF consumption was associated with the increased risk of diabetes by 15% in adults after adjusted for potential socioeconomic and lifestyle factors [13], while a cross-sectional study found among Brazil’s pregnant women that UPF intake was not associated with gestational diabetes mellitus [14]. There is no investigation of UPF intake and diabetes yet in China. 

Despite the emerging evidence of UPF and its association with health risks, the consumption of the poor-quality food has been increasing in line with the economic development and urbanization, especially in nutrition transition countries (e.g., India, Indonesia, and Brazil) [15]. Studies has shown that food choice is based not only on nutrients profile but also on the taste, convenience and cost [16] which may partly drive the trend. 

China had experienced a remarkable nutrition transition in the past several decades. Diet changed from dominantly a traditional pattern of home-made food out of natural food sources towards a modern one of increased processed food and drink packs from supermarket [17] that is associated with cardiometabolic risks [18]. We recently reported using national representative data from China Nutrition and Health Survey (CNHS) that UPF consumption per capita was increased fourfold during 1997–2011 among Chinese adults aged over 20 years and higher UPF consumption was associated with overweight/obesity [19]. However, its long-term association with diabetes and the impact of overweight/obesity on the association have not been investigated in this population. We aimed to fill the knowledge gap among adults attending the CHNS.

## 2. Materials and Methods

### 2.1. Study Design and Sample 

This is an association study between repeated measurements of dietary intake and diabetes status during 1997–2011 using public access CHNS data.

**Figure 1:**
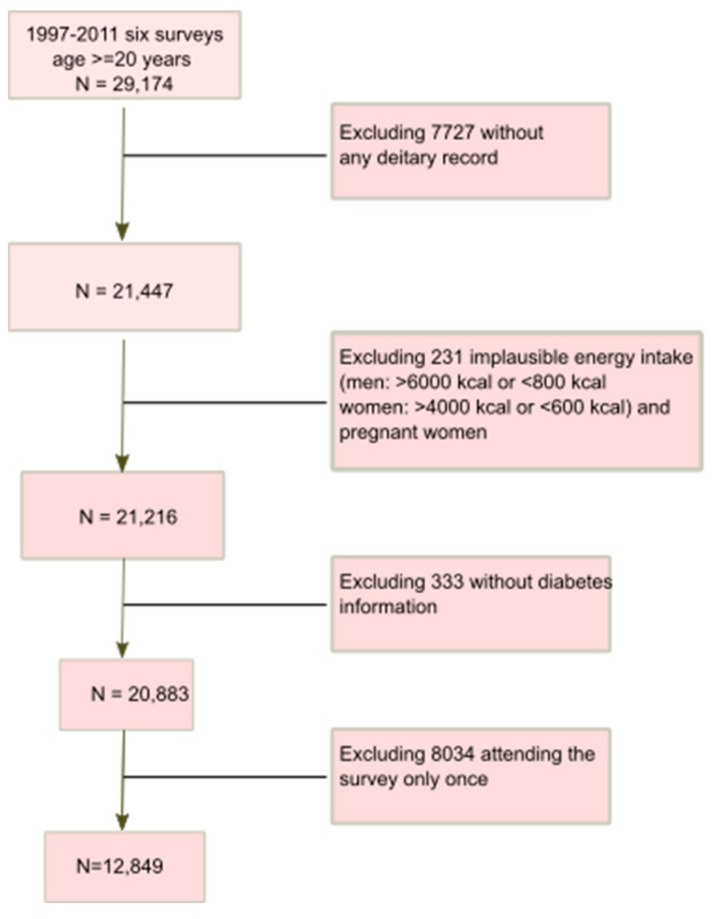
Sample flowchart of participants attending CHNS 1997−2011.

The CHNS study was a continuing open household-based cohort study conducted in nine provinces in China [20]. Samples in both urban and rural areas were drawn by a multistage random-cluster sampling method. So far, ten waves of dietary data collection have been completed (1989, 1991, 1993, 1997, 2000, 2004, 2006, 2009, 2011 and 2015). Blood samples were collected in the 2009 and 2015 surveys. However, blood glucose data in 2015 were not open to the public. The overall response rate was >60% based on the first survey in 1989 and >80% based on the previous year [20]. In this study, a total of 12,849 eligible adults were included based on the following criteria: aged ≥ 20 years; having self-reported diagnosis of diabetes and/or fasting blood tests; having attended at least two nutrition surveys during 1997–2011; having plausible energy intake (800–6000 kcal/d for men, and 600–4000 kcal/d for women) (Figure 1). Informed consent was obtained from all participants. The survey was approved by the institutional review committees [20]. 

### 2.2. Outcome Variable

The primary outcome was diabetes. Diabetes was self-reported at each survey during 1997–2011. It was ascertained if a participant answered yes to either of the following questions: “Has the doctor ever told you that you suffer from diabetes?” “if yes, How old were you when the doctor told you about such a situation” “Did you use any of the treatment methods for diabetes (for example, on diet, weight control, oral medicine, Injection of insulin, Chinese, home remedies, Qigong)?”. In addition, fasting plasma glucose was obtained in 2009 with diabetes defined as fasting plasma glucose ≥ 7.0 mmol/L, HbA1c ≥ 48 mmol/mol (equivalent to 6.5%). Diabetes in 2009 was ascertained if a participant self-reported being told having diabetes, or if self-reported not been told having diabetes but blood tests results met the diagnostic criteria. Fasting blood was taken in the morning and prepared for a further test in a national central lab in Beijing (medical laboratory accreditation certificate ISO 15189: 2007). Fasting plasma glucose was measured with the GOD-PAP method (Randox Laboratories Ltd., Crumlin, UK). All the measurements and tests were collected using standard protocol by trained staff. The detailed data collection protocol was described elsewhere [20].

### 2.3. UPF Assessment

At each survey, individual dietary intake was collected by a trained investigator conducting a 24-h dietary recall on each of 3 consecutive days [21]. Foods and condiments in the home inventory, foods purchased from markets or picked from gardens, and food waste were weighed and recorded by interviewers at the beginning and end of the three-day survey period. The types and amount of food, the type of meal and the place of consumption for a participant were from both dietary recall and the records kept by the individual. Cooking oil and condiments consumption for everyone in the household was estimated using individual energy-weighted intake. Detailed description of the dietary measurement has been published previously [22]. The food intake data in 1997–2011 was recoded and converted to nutrient intake using the corresponding updated food composition tables [23]. Around 3000 food items in the food composition tables since 1997 were categorized into four groups by the NOVA classification [6,19]. Long-term cumulative mean UPF intake at each survey was calculated from all the proceeding surveys to reduce within individual variation. For instance, if the UPF intake of a participant was a, b, c in 1997, 2004, and 2009, the corresponding mean UPF intake in 1997, 2004 and 2009 was a, (a + b)/2, and (a + b + c)/3.

### 2.4. Covariates 

Sociodemographic and lifestyle factors were collected at each survey using a structured questionnaire. The socioeconomic status included: education (low: illiterate/primary school; medium: junior middle school; high: high middle school or higher), annual family income (recoded into tertiles as low, medium and high), urbanization levels (recoded into tertiles as low, medium and high).

Height, weight, and blood pressure were measured at each survey round. Overweight/obesity was defined as BMI ≥ 25 kg/m^2^. Hypertension was defined as systolic blood pressure ≥ 140 mmHg and/or diastolic blood pressure ≥ 90 mmHg or having known hypertension.

Physical activity level (metabolic equivalent of task) was estimated based on self-reported activities and duration using a Compendium of Physical Activities. Smoking status was categorized as non-smokers, ex-smokers and current smokers. Alcohol consumption was recorded as yes or no. Two dietary patterns (traditional and modern) were identified in this population using principal components analysis from thirty-five food groups of similar nutrient profiles or culinary uses [18]. The traditional one was characterized by high intakes of rice, meat, and vegetables, while the modern pattern was highly correlated with fast food, milk, and deep-fried food [18]. 

### 2.5. Statistical Analysis 

Mean UPF intake was grouped into: non-consumers, 1–19, 20–49, and ≥50 g/day based on that the serving size in the context of Chinese food is *Liang* (50 g). Sample characteristics were presented and compared by UPF intake levels using ANOVA for continuous measures or chi-square tests for categorical ones. 

The association between UPF intake and diabetes were assessed using mixed effect logistic regression models. Unadjusted and adjusted odds ratios (95% CI) of the fixed part of the models were reported. Adjusted models were built by including age, sex, and energy intake initially in Model 1; further adding fat intake, socioeconomic status (income, urbanization, and education), and lifestyle factors (smoking, alcohol drinking, and physical activity) in Model 2, and next adjusted for overweight/obesity or hypertension in Model 3 or Model 4. Model 5 included BMI, hypertension, and dietary patterns; Sensitivity analysis was presented as Model 6 from Model 5 among participants attended at least four waves of the surveys (*n* = 7263).

A subgroup analysis was conducted among 8382 participants in 2009 with self-report diagnosis of diabetes and/or fasting blood records. The association between UPF intake in 1997–2009 or in 2009 and diabetes was assessed using logistic regression analysis.

To test the interaction between UPF intake and other covariates (sex, sociodemographic, lifestyle, diet, and health factors), a product term of these two variables was put in the regression model. 

The analyses were performed using STATA 17.0 (Stata Corporation, College Station, TX, USA). Statistical significance was considered when *p* < 0.05 (two-sided).

## 3. Results

### 3.1. Population Profile

At entry, the mean age of the participants was 43.3 years old (SD 14.8). In total, 49.0% were men, one third had medium level of income or lived in high urbanized areas, 44.9% attained low level of education, more than 30% were current smokers or alcohol drinkers. The prevalence of hypertension and diabetes were 15.9% and 2.1%, respectively. The percentages of UPF energy over total energy intake for non-consumers, 1–19 g/d, 20–49 g/d, and ≥50 g/d were 0, 1.6%, 4.9%, 14.3%. And the corresponding weight percentages of UPF over total food intake in gram per day were 0, 1.2%, 3.2%, and 10.4%.

### 3.2. Consumption of UPF during 1997–2011

The mean UPF consumption (age- and sex-adjusted) increased continuously from 12.6 g/day in 1997 to 41.3 g/day in 2011 with sharp increase since 2004. The daily energy contribution of UPF increased from 1.4% in 1997 to 4.9% in 2011 and the daily food weight proportion of UPF from 1.1% to 3.6%. At entry, 11% (*n* = 1396) of the participants had UPF intake greater than 50 g/d. 

Compared to those with no or lower UPF intake of 1–19 g/d, participants having UPF ≥ 50 g/d at entry were more likely: being males, or having higher level of education or income, or living in the higher urbanized areas, or being smokers or alcohol drinkers, or having higher BMI. Energy, fat, protein intakes, and modern dietary pattern score were higher, while intake of carbohydrate and traditional dietary pattern score were lower (Table 1).

### 3.3. Diabetes and UPF Consumption Level

The prevalence of diabetes increased eight times from 1.5% in 1997 to 11.2% in 2009 and to 12.1% in 2011. The unadjusted ORs (95% CI) of diabetes for UPF consumption levels of none, 1–19 g/d, 20–49 g/d, and >50 g/d were 1 (reference), 2.13 (1.76, 2.56), 2.79 (2.29, 3.40), and 2.60 (2.10, 3.23), respectively (*p* < 0.001). The odds ratios remained significant after adjusted for age, sex, and energy intake (aOR 2.21; 95% CI 1.76, 2.77 for ≥50 g/d Model 1) and after further adjusted for fat, behavioural and sociodemographic factors (aOR 1.96; 95% CI 1.53, 2.51 in Model 2). Adjusted for either BMI or hypertension did not change the relative odds substantially in Model 3 or Model 4. Nor did BMI and hypertension, and overall dietary patterns. Specifically, the aORs (95% CI) of diabetes for UPF level of 20–49 g/d and ≥50 g/d were 1.49 (1.19–1.86), 1.40 (1.08–1.80), respectively. Sensitivity analysis among participants attending four waves of the surveys showed the corresponding aORs (95% CI) of 1.55 (1.20–2.00) and 1.37 (1.00–1.88) (Table 2).

The cross-sectional analysis of 8382 participants in 2009 showed both UPF intake in 1997–2009 or in 2009 was positively associated with diabetes. After adjusted for sociodemographic and lifestyle factors, the ORs (95% CI) of diabetes for UPF intake of 1–19 g/d, 20–49 g/d, and ≥50 g/d were 1.05 (0.86–1.28), 1.21 (0.96–1.51), and 1.31 (1.04–1.65) (*p* for trend = 0.015), respectively, compared with no UPF intake. Similarly, BMI slightly attenuated the association. The cross-sectional association using UPF intake in 2009 showed the corresponding adjusted ORs (95% CI) were 1.16 (0.79–1.68), 0.85 (0.62–1.15), and 1.23 (1.01–1.50) (*p* for trend = 0.037) (Table 3). 

The association was consistent across subgroups by sex, education, income, urbanization, smoking, overweight/obesity, and hypertension status (Table 4).

## 4. Discussion

Among the 12,849 participants in the CHNS, the mean per capita UPF consumption increased from 12.6 g/day in 1997 to 41.3 g/day in 2011 and the UPF contribution to daily total energy or daily total foods rose from 1.4 to 4.9% or 1.1 to 3.6%. Meanwhile, the prevalence of diabetes increased eight times from 1.5 to 12.1% in 2011. UPF intake ≥ 50 g/d increased the risk of diabetes by 40% compared with non-consumers. 

Although the per capita UPF consumption and proportion of diet weight in China was below the level observed in other countries [8] and it is impossible to compare directly due to different UPF items, methodology and study period among these studies, it is unquestionable that the increased trend in China was dramatic, especially among those who were younger, or had higher educational attainment, or resided in highly urbanized areas. The younger people were more likely to eat out compared to older adults in China as home-prepared food are of better quality [24] while eating out increased the consumption of UPF by 41% compared with preparing meals exclusively at home [25]. The subgroup had higher educational levels and lived in highly urbanized area facilitating UPF consumption for time saving, savory taste, attractive packaging, and affordability [26].

The association between UPF and diabetes among this Chines population was consistent with the synthesized result of observational studies among adults in France, Netherland, UK, Spain, and Canada [13]. All studies applied the NOVA classification and four of them had follow-ups of 3.4–12 years [27,28,29,30] with HR/OR ranging from 1.13 to 1.53. The Canadian cross-sectional survey data reported 37% increased odds of self-reported diabetes in 2015 [31] while our result using UPF consumption data in 2009 reported the increased odds of 23% for diabetes. 

Potential mechanisms underlying the association should be noted. Studies had shown that UPF was rich in added/free sugars and saturated fats, which were positively associated with diabetes [32,33]. Grains, meat, vegetables, and fruits lost the physical and structural characteristics of the food matrix during processing, which would result in a high glycaemic index [34]. In addition, as satiety mechanisms showed, humans are more sensitive to volume than energetic content [35], therefore, UPF with higher energy density may facilitate excessive energy intakes, leading to obesity as showed in our previous study [36]. We found in this study that obesity attenuated partly the association between UPF and diabetes. This is supported by a follow up study indicating a 23% increased risk of incident diabetes with each kg/m^2^ increase in BMI (95% CI 1.22 to 1.24) among 211, 833 Chinese persons >20 years old across 32 sites and 11 cities in China [37].

Food additives in UPF should not be ignored since the association was independent of energy and fat intake. More than 2000 food additives in 23 different categories have been added during food processing in China [38]. Although a maximum dose limitation for each additive has been set, it’s unknown whether the long-term intake of these safe-dose food additives, whether single or combined, has cumulative or synergetic adverse effects on health. Emerging evidence has suggested that very low concentrations of polysorbate 80, the common food emulsifier, might change the gut microbiota, increase bacterial translocation, cause intestinal inflammation and promote type 2 diabetes [39]. Exposure to Carrageenan would result in glucose intolerance and fasting hyperglycaemia [40]. Sucralose, as a non-caloric artificial sweetener, could alter the metabolic response to the glucose load and slow down insulin clearance from plasma [41]. Furthermore, heat treatment during food processing, in particularly, could pose exposure to contaminants such as acrylamide which was associated with insulin resistance [42]. Finally, UPF could be contaminated by the package material with endocrine-disrupting properties (e.g., bisphenols A) [43] in order to keep within the extended expiration date. The impact of food additives on health and food processing in China should be closely regulated and monitored. 

To our best knowledge, this is the first association study between long-term UPF consumption and diabetes in Chinese adults. The use of mean UPF intake during 1997–2011 from 3-day dietary intake in combination with household food inventory provided a robust estimate of long-term habitual intake. An updated NOVA classification system was used to classify UPF in this population. The association was confirmed by sensitivity analysis. Potential confounding factors including sociodemographic, behavioural, health, and dietary factors were adjusted. 

Limitations should not be ignored. First, misclassification was possible due to lack of completing information about food processing in the CHNS survey that was not specifically designed for NOVA classification. Second, we used the weight unit (gram) to estimate the consumption of UPF which might not be precise for the diverse UPF items. Third, due to the complexity of food processing and variabilities in additive composition between brands for a similar type of product, we could only roughly group some food items therefore the association could be biased. The NOVA classification has been criticized on its lack of specificity at an individual nutrient level or overall adequacy of dietary patterns [44]. We have incorporated both nutrients and dietary pattern in the study to overcome the pitfalls. Fourth, the ascertainment of diabetes was self-reported except for 2009 which might pose misclassification of the outcome. However, out subgroup analysis using diabetes identified in 2009 and UPF intake either in 1997–2009 or in 2009 showed consistent results. Also, the prevalence and the temporal trend of diabetes in the study period were consistent with other national estimates [45,46,47], especially the prevalence in 2009 when both self-report and blood tests were applied to ascertain diabetes. This study did not distinguish between type 1 and type 2 diabetes. The association was unlikely to change much, as the data showed among the 1219 participant self-reported having been told to have diabetes in 1997–2011, only 30 cases (2.5%) were identified at the age of under 20. In addition, population-based data indicates Type 1 diabetes onset peak is in the 10–14-year-old age group in Chinese population [48]. Finally, residual confounding was still possible, for example, there was no record on family history of diabetes and ethnicity which is closely related to culinary culture in China. 

## 5. Conclusions

Both UPF consumption and the prevalence of diabetes increased during 1997–2011 in Chinese adults. Higher UPF consumers had a significantly higher risk of diabetes than non-consumers. The association between UPF consumption and diabetes was partly mediated by overweight/obesity. In facing with the diabetes epidemic in China, nutrition education should focus on in part the modification of the unhealthy dietary factor and the maintenance for healthy weight. 

## Figures and Tables

**Table 1:** Sample characteristics by ultra-processed food intake among participants attending China Health and Nutrition Survey (*n* = 12,849).

	None	1–19 g/d	20–49 g/d	≥50 g/d	*p*-Value
**N**	***n* = 10,129**	***n* = 616**	***n* = 708**	***n* = 1396**	
**Survey year at entry**					<0.001
1997	58.7%	63.6%	48.2%	39.9%	
2000	16.4%	13.3%	15.3%	15.9%	
2004	12.4%	11.5%	12.9%	12.0%	
2006	5.0%	3.4%	9.9%	9.7%	
2009	7.5%	8.1%	13.8%	22.6%	
**Age, mean (years)**	43.2 (14.7)	43.7 (15.9)	43.2 (15.2)	44.2 (14.7)	0.091
**Sex**					<0.001
Men	46.8%	44.2%	50.8%	66.0%	
Women	53.2%	55.8%	49.2%	34.0%	
**Income**					<0.001
Low	31.7%	24.2%	20.3%	21.8%	
Medium	33.2%	34.5%	32.2%	31.6%	
High	35.0%	41.3%	47.5%	46.7%	
**Education**					<0.001
Low	47.5%	42.6%	30.9%	33.5%	
Medium	32.0%	33.0%	32.7%	30.8%	
High	20.4%	24.4%	36.4%	35.7%	
**Urbanization**					<0.001
Low	36.4%	28.2%	19.8%	20.5%	
Medium	30.1%	26.9%	25.3%	28.2%	
High	33.5%	44.8%	54.9%	51.4%	
**Energy intake, mean (kcal/d)**	2242.9 (633.1)	2153.6 (595.6)	2214.7 (600.3)	2480.9 (702.5)	<0.001
**Percent (%) of UPF over total energy intake/d**	0.0 (0.0)	1.6 (1.5)	4.9 (2.8)	14.3 (11.1)	<0.001
**Percent (%) of UPF over total food intake/d**	0.0 (0.0)	1.2 (0.7)	3.2 (1.2)	10.4 (6.8)	<0.001
**Fat intake, mean (g/d)**	65.3 (35.7)	65.3 (33.7)	75.6 (35.7)	82.1 (39.9)	<0.001
**Protein intake, mean (g/d)**	67.5 (22.1)	67.5 (21.7)	71.1 (22.9)	76.6 (25.0)	<0.001
**Carbohydrate intake, mean (g/d)**	345.7 (120.6)	322.2 (114.2)	308.7 (112.0)	323.1 (113.6)	<0.001
**Traditional dietary pattern score, mean**	−0.0 (1.0)	0.1 (0.9)	0.1 (1.0)	0.1 (1.0)	<0.001
**Modern dietary pattern score, mean**	−0.3 (0.7)	−0.2 (0.8)	0.2 (1.0)	0.7 (1.2)	<0.001
**Smoking**					<0.001
Non smoker	69.1%	69.8%	66.1%	53.5%	
Ex-smokers	1.3%	1.0%	2.1%	3.0%	
Current smokers	29.6%	29.3%	31.8%	43.5%	
**Alcohol drinking**	32.1%	34.5%	39.8%	57.8%	<0.001
**Physical activity, mean (MET-hrs/week)**	141.0 (117.0)	135.6 (117.2)	132.2 (112.5)	143.1 (118.9)	0.15
**BMI (kg/m^2^), mean (SD)**	22.6 (3.2)	22.8 (3.3)	23.1 (3.3)	23.0 (3.3)	<0.001
**Diabetes**	2.0%	1.3%	2.5%	2.8%	0.087
**Hypertension**	15.1%	19.4%	16.5%	19.5%	<0.001

Data in table as *n* (%) or mean (SD). *p* values from ANOVA or chi square test.

**Table 2:** Odds ratio (95% CI) for diabetes by cumulative ultra-processed food intake in 1997–2011 among participants attending China Health and Nutrition Survey.

	Cumulative UPF Intake (g/day)				
	None	1–19	20–49	≥50	*p* for Trend
Unadjusted	1.00	2.13 (1.76–2.56)	2.79 (2.29–3.40)	2.60 (2.10–3.23)	<0.001
Model 1	1.00	1.53 (1.27–1.85)	2.15 (1.76–2.64)	2.21 (1.76–2.77)	<0.001
Model 2	1.00	1.34 (1.09–1.65)	1.87 (1.50–2.34)	1.96 (1.53–2.51)	<0.001
Model 3	1.00	1.29 (1.05–1.58)	1.79 (1.43–2.23)	1.85 (1.45–2.36)	<0.001
Model 4	1.00	1.29 (1.05–1.58)	1.74 (1.40–2.17)	1.79 (1.40–2.29)	<0.001
Model 5	1.00	1.21 (0.98–1.48)	1.49 (1.19–1.86)	1.40 (1.08–1.80)	<0.001
Model 6	1.00	1.22 (0.97–1.53)	1.55 (1.20–2.00)	1.37 (1.00–1.88)	0.003

Odds ratios from mixed effect logistic regression. Model 1: adjusted for age, gender and energy intake. Model 2: model 1further adjusted for intake of fat, income, urbanicity, education, smoking, alcohol drinking, and physical activity. Model 3: model 2 further adjusted hypertension. Model 4: model 2 further adjusted BMI. Model 5: model 2 further adjusted hypertension, BMI and dietary patterns [18]. Model 6: model 5 among all participants who attended at least four waves of survey (*n* = 7263).

**Table 3:** Odds ratio (95% CI) for diabetes by cumulative ultra-processed food intake among participants attending China Health and Nutrition Survey in 2009 (*n* = 8382).

	None	1–19 g/d	20–49 g/d	≥50 g/d	*p* for Trend
	*n* = 3764	*n* = 1947	*n* = 1323	*n* = 1348	
Diabetes cases	364	227	169	180	<0.001
Unadjusted	1.00	1.23 (1.03–1.66)	1.37 (1.13–1.66)	1.44 (1.19–1.74)	0.003
Model 1	1.00	1.10 (0.92–1.31)	1.27 (1.04–1.55)	1.46 (1.19–1.77)	<0.001
Model 2	1.00	1.05 (0.86–1.28)	1.21 (0.96–1.51)	1.31 (1.04–1.65)	0.015
Model 3	1.00	1.07 (0.87–1.31)	1.16 (0.92–1.46)	1.24 (0.97–1.57)	0.060
Sensitivity analysis	1.00	1.16 (0.79–1.68)	0.85 (0.62–1.15)	1.23 (1.01–1.50)	0.037

Odds ratio from logistic regression analysis using diabetes in 2009 as outcome and UPF intake in 1997–2009 as study factor. Model 1: adjusted for age, gender and energy intake. Model 2: Model 1 further adjusted for intake of fat, smoking, alcohol drinking, income, urbanicity, education, physical activity, intake of fruit and vegetable. Model 3 further adjusted for BMI. Sensitivity analysis: model 2 among those with UPF intake in 2009 (*n* = 8382).

**Table 4:** Stratified analysis of the association between cumulative UPF consumption in 1997–2011 and diabetes by sample characteristics.

	Cumulative UPF Intake (g/day)					
	None	1–19	20–49	≥50	*p* Value	*p* Interaction
**Sex**						0.637
Men	1.00	1.51 (1.10–2.09)	2.00 (1.45–2.76)	2.22 (1.60–3.09)	<0.001	
Women	1.00	1.23 (0.94–1.61)	1.82 (1.34–2.49)	1.78 (1.21–2.61)	<0.001	
**Education**						0.146
Low	1.00	1.59 (1.21–2.10)	2.62 (1.90–3.61)	2.22 (1.50–3.30)	<0.001	
Medium	1.00	0.95 (0.63–1.45)	1.51 (0.98–2.32)	1.77 (1.11–2.80)	0.008	
High	1.00	1.03 (0.65–1.63)	1.12 (0.70–1.79)	1.39 (0.86–2.25)	0.195	
**Income**						0.475
Low	1.00	1.14 (0.80–1.64)	2.25 (1.50–3.37)	2.11 (1.32–3.38)	<0.001	
Medium	1.00	1.25 (0.89–1.76)	1.40 (0.94–2.10)	1.56 (1.00–2.45)	0.020	
High	1.00	1.34 (0.94–1.91)	1.99 (1.38–2.85)	2.02 (1.37–2.98)	<0.001	
**Urbanization**						0.469
Low	1.00	1.12 (0.71–1.77)	1.34 (0.73–2.45)	1.36 (0.69–2.68)	0.223	
Medium	1.00	1.45 (1.00–2.11)	1.88 (1.24–2.83)	2.49 (1.61–3.85)	<0.001	
High	1.00	1.24 (0.93–1.67)	1.99 (1.45–2.71)	1.81 (1.27–2.57)	<0.001	
**Smoking**						0.987
Non smoker	1.00	1.37 (1.08–1.73)	1.99 (1.52–2.61)	2.18 (1.61–2.97)	<0.001	
Current smokers	1.00	1.29 (0.86–1.93)	1.56 (1.04–2.35)	1.68 (1.11–2.52)	0.006	
**Overweight/obesity**						0.366
No	1.00	1.14 (0.85–1.53)	1.77 (1.29–2.43)	1.70 (1.19–2.41)	<0.001	
Yes	1.00	1.43 (1.06–1.92)	1.72 (1.24–2.38)	1.91 (1.35–2.71)	<0.001	
**Hypertension**						0.684
No	1.00	1.19 (0.90–1.56)	1.61 (1.19–2.16)	1.77 (1.28–2.43)	<0.001	
Yes	1.00	1.34 (1.00–1.79)	1.94 (1.41–2.66)	1.87 (1.32–2.66)	<0.001	

Odds ratio (95% CI) from mixed effect logistic regression. Model adjusted for age, sex, intake of energy and fat, education levels, income, urbanization, smoking, alcohol drinking, and physical activity.

## Data Availability

The current research uses data from the China Health and Nutrition Survey (CHNS). Data described in the manuscript, code book, and analytic code are made publicly and freely available without restriction at https://www.cpc.unc.edu/projects/china accessed on 15 January 2019.
